# Risk factors for amputation in patients with diabetic foot ulcer in southwest Iran: a matched case-control study

**DOI:** 10.4178/epih/e2015044

**Published:** 2015-10-05

**Authors:** Mohammad Kogani, Mohammad Ali Mansournia, Amin Doosti-Irani, Kourosh Holakouie-Naieni

**Affiliations:** Department of Epidemiology and Biostatistics, School of Public Health, Tehran University of Medical Sciences, Tehran, Iran

**Keywords:** Amputation, Risk factors, Case-control studies, Diabetes mellitus, Iran

## Abstract

**OBJECTIVES:**

Amputation is a multifactorial complication in diabetic patients. The aim of this study was to determine the risk factors associated with amputation in patients with diabetic foot ulcers.

**METHODS:**

This matched case-control study was conducted based on new cases of amputation from March 2012 to November 2014. We selected new cases who had undergone amputation, and the control group was chosen from the cities or areas where the cases resided. Each case was matched with two controls based on the duration of diabetes and location. Conditional logistic regression was used to evaluate the associations between potential risk factors and amputation.

**RESULTS:**

A total of 131 cases were compared with 262 controls. The results of the adjusted model showed that sex (odds ratio [OR], 8.66; 95% confidence interval [CI], 2.68 to 27.91), fewer than two hemoglobin A1c (HbA1c) tests per year (OR, 13.97; 95% CI, 4.97 to 39.26), unsuitable shoes (OR, 5.50; 95% CI, 2.20 to 13.77), smoking (OR, 3.44; 95% CI, 1.45 to 8.13), and body mass index (OR, 1.20; 95% CI, 1.03 to 1.41) were associated with amputation in diabetic patients.

**CONCLUSIONS:**

The most important factors associated with amputation were females, irregular monitoring of HbA1c levels, improper footwear, and smoking. Developing educational programs and working to ensure a higher quality of care for diabetic patients are necessary steps to address these issues.

## INTRODUCTION

Amputation is the process of removing part of the body, usually a limb, and is often performed in the lower extremities of the body. Diabetes is the major factor leading to amputation in non-traumatic cases (5). Epidemiological studies have shown that each year, 2.5% of patients with diabetes are affected by diabetic foot ulcers, and that 15% of patients with diabetes will ultimately be affected by diabetic foot ulcers [1,2]. In recent years, the annual prevalence of diabetes has increased by approximately six percent, to the point that the world is now facing a diabetes pandemic. Current estimates suggest that there are approximately 200 million diabetes patients throughout the world. According to the World Health Organization, 300 million adults will have diabetes in 2025 [3-6]. Diabetes causes a range of complications, such as nephropathy, retinopathy, neuropathy, diabetic foot ulcers, and cardiovascular disease, and the incidence of complications is expected to increase with the rising number of cases of diabetes [7-9]. In many countries, complications of diabetes are the most important cause of blindness, amputation, and kidney failure among people aged 20 years to 70 years old [10]. Foot ulcers are the most common complication of diabetes that is frequently overlooked; if neglected, diabetic foot ulcers can ultimately lead to amputation [11]. Diabetic foot ulcers and amputation are acute health and socioeconomic problems that negatively affect the quality of life of patients and impose a high economic burden on the patients and society [12].

Of patients with foot ulcers, 20% to 50% eventually undergo amputation [13-15]. Diabetic patients have a 15 to 20 times higher risk of amputation than non-diabetic patients [11,16,17].

Amputation is a multifactorial complication in diabetic patients. Older age, being male, and the duration of disease have been reported to be risk factors for amputation [11]. In various studies, the incidence of amputation in diabetic patients has been reported to range from 5.2% to 39.4% [2,11,13]. These strikingly different estimates of incidence may reflect variation in the risk factors present in different populations. Thus, detection and control of these risk factors can largely prevent the occurrence of amputation and its consequences. In Western countries, several reports have addressed the risk factors for amputation [1], but in Iran, few studies have been conducted on this topic. Due to the debilitating effects of amputation, the fact that it is relatively understudied in Iran, and the existence of gaps in the prevention and treatment of amputation, it is necessary to conduct a study to further evaluate the risk factors for amputation among Iranian diabetic patients. The aim of this study was to determine the risk factors for amputation in patients with diabetic foot ulcers in southwest Iran.

## MATERIALS AND METHODS

A matched case-control study was designed and carried out in Razi Hospital (the reference hospital for patients with diabetic foot ulcers) in Ahvaz, a city located in the southwest of Iran in the Khuzestan province. The research council of the School of Public Health of the Tehran University of Medical Science approved this study.

The case group included new diabetic patients who had been referred to Razi Hospital and undergone amputation since March 2012. The case selection process continued until a sample size of 131 individuals was obtained. The control group members were chosen from the cities or areas where the cases lived, and were selected randomly from the records of appropriate patients in the corresponding health centers.

In order to adjust for confounding variables more effectively and increase the precision of the study, the cases and controls were matched according to the duration of diabetes. Matching was carried out via the caliper matching method; accordingly, the length of the continuous variable was considered to be ± two years. In order to increase the power of our study, we selected two controls for each case. The records of patients admitted to the hospital were reviewed in order to identify patients who had undergone amputation. Finally, 131 patients with diabetic foot ulcers who had undergone amputation were included in the study. After obtaining informed consent from each patient, we conducted an interview to collect the required data using a predesigned questionnaire.

The case group members resided in different cities in the Khuzestan province at the time of the occurrence of diabetic foot ulcers and amputation. Therefore, in order to avoid possible bias and to achieve maximal matching in terms of socioeconomic status and access to healthcare facilities, the control group was randomly selected from all diabetic patients without diabetic foot ulcers who lived in the same city as each case.

The data collection tool was a predesigned questionnaire that included demographic, clinical, and epidemiological data. The quantitative data from this study were examined and presented as mean and standard deviation (SD). The qualitative data were presented as frequency and percentage. The relationships between different variables and amputation were analyzed using conditional logistic regression, and the association between each variable and amputation was expressed as an odds ratio (OR) with a 95% confidence intervals (CIs).

A conditional logistic regression model was used to assess the associations between potential risk factors and amputation. In the process of model building, variables with a p-value <0.2 in the univariate analysis were first entered into the multiple regression model. In the next step, we removed risk factors that lost their significance. The variables that were excluded based on the univariate analysis were then entered into the model to determine whether adding them to the multivariate model led to important changes. The likelihood ratio test was used to select the best model. Fractional polynomials were used to model the continuous variables [18]. STATA version 11 (Stata Corp., College Station, TX, USA) was used for the statistical analysis.

## RESULTS

A total of 131 cases (patients who underwent amputation) and 262 controls were included in this study. Among the cases, 91 of the participants (69%) were female, while in the control group, 121 (46.18%) were female.

The mean (SD) ages of the cases and controls were 66.16 years (8.16 years) and 62.58 years (8.14 years), respectively. Among the cases, 21 patients (16.03%) had undergone a hemoglobin A1c (HbA1c) test twice or more each year, compared to 172 patients (65.65%) in the control group. A total of 96 cases (73.28%) had a history of smoking, compared to 98 individuals (37.40%) in the control group. Moreover, 94 patients (71.76%) in the case group and 122 patients (46.56%) in the control group had a close family member with a history of diabetes.

In the case group, 79 patients (60.31%) had a history of taking insulin, compared to 100 people (38.17%) in the control group. In the case group, 25 patients (19.8%) had suitable footwear, compared to 151 patients (57.61%) in the control group (Table 1).

The unadjusted conditional logistic regression analysis showed a direct association between age and amputation (OR, 1.08; 95% CI, 1.04 to 1.12). The odds ratio for amputation in female patients compared to male patients was 2.62 (95% CI, 1.66 to 4.11). Each 1 kg/m2 increase in body mass index (BMI) was associated with a 1.15-fold increase in the odds of amputation (95% CI, 1.06 to 1.24). An association was found between the insulin therapy and amputation (OR, 0.36; 95% CI, 0.23 to 0.58). The OR for each one-year increase in the duration of insulin therapy was 1.27 (95% CI, 1.15 to 1.40). The OR for amputation was 8.5 (95% CI, 4.8 to 15.04) in patients who monitored their HbA1c levels less than twice per year. Smoking was associated with an OR for amputation of 4.26 (95% CI, 2.64 to 6.87), showing that the odds of amputation in people who smoked were 4.26 times higher than in those who did not smoke. The OR for amputation was 0.34 (95% CI, 0.21 to 0.55) in patients with no history of diabetes among their close family members. Unsuitable shoes were associated with an OR for amputation of 4.75 (95% CI, 2.91 to 7.76), showing that the likelihood of amputation in people who did not have proper shoes was 4.75 times higher than in those who had suitable shoes. The OR for patients with hypertension was 1.26 (95% CI, 0.82 to 1.92), however this association was not statistically significant (Table 1).

According to the multiple conditional logistic regression analysis, the adjusted OR for amputation was 8.66 (95% CI, 2.68 to 27.91) in female patients in comparison with male patients. Monitoring HbA1c levels less than twice annually was significantly associated with amputation, with an adjusted OR of 13.97 (95% CI, 4.97 to 39.26). The adjusted OR for amputation associated with a lack of suitable shoes was 5.50 (95% CI, 2.20 to 13.77). Compared to non-smokers, the adjusted OR for amputation among smokers was 3.44 (95% CI, 1.45 to 8.13). Higher BMI values were directly associated with amputation (OR, 1.20; 95% CI, 1.03 to 1.43). A family history of amputation increased the OR of amputation by 3.96 (95% CI, 1.49 to 10.53) compared to those without a family history of amputation (Table 2).

## DISCUSSION

According to the results of our unadjusted analysis, amputation was directly associated with age, sex, the number of years of insulin therapy, the lack of suitable shoes, higher BMI values, smoking, a history of diabetes among close family members, and irregular HbA1c monitoring. Contrastingly, an association was found between amputation and the insulin therapy. Multiple conditional logistic regression indicated that the most important predictors of amputation in diabetic patients were sex, infrequent HbA1c monitoring, the lack of suitable shoes, smoking, BMI, a family history of amputation, and employment status. In addition, a significant relationship was found between the odds of amputation and having unsuitable footwear; in fact, the odds of amputation in patients with inappropriate shoes were 5.5 times higher than in patients with appropriate shoes. This finding of our study is consistent with the results of another study [19], which concluded that the selection of appropriate footwear is essential for diabetic patients, especially those with neuropathy. The findings of this study indicate that using suitable shoes was an important factor in preventing amputation in Iranian diabetes patients. This finding also underscores the importance of physicians and/or nurses educating diabetic patients regarding protection of their feet through wearing suitable shoes.

In our study, 68% of the patients who underwent amputation were female. The relationship between the female and amputation was significant both in the unadjusted and adjusted models. Females were found to be at an 8.66 times higher risk of amputation than males. This result is consistent with the findings of another study [20], but is inconsistent with the results of Mashaekhi et al. [21], who conducted a cross-sectional study evaluating the prevalence of amputations in patients with diabetic foot ulcers and found a significant relationship between males and risk of amputation. This discrepancy is likely due to differences in the methods of these studies, since our study was a case-control study, whereas their study was a cross-sectional study. However, the cross-sectional study performed by Nikkhooy et al. [22] found no significant relationship between sex and amputation. Considering these discrepancies, it seems necessary to conduct more research in this area.

We assessed the relationship between annual monitoring of HbA1c levels and the odds of amputation in diabetic patients. In our study, this variable was found to be one of the most important factors that had a significant relationship with amputation. According to the results of the multivariate conditional logistic regression analysis, patients who monitored their HbA1c levels less than twice per year were at a 13.97 times higher risk of amputation than those who monitored their status twice or more each year. This finding is consistent with the results of another study conducted in Germany in 2012 [11]. In that study, Kaplan-Meier survival analysis showed that amputation was independently associated with higher HbA1c values, and the hazard ratio for amputation associated with an HbA1c value >7.5% was 1.20. Our estimate of the OR of infrequent monitoring of HbA1c for amputation (OR, 13.97) was strikingly high, which may have been due to unknown confounders that we did not assess. Therefore, we recommend that future studies evaluate the association between infrequent HbA1c monitoring and amputation. HbA1c monitoring is a screening tool for diabetic patients that shows how successful patients have been in controlling their disease [23], meaning that monitoring HbA1c levels frequently can help patients and their physicians detect diabetic complications relatively early. Patients who regularly checked their HbA1c levels over the course of a year were probably healthier than those who did not, meaning that regular HbA1c screenings might be effective in reducing the risk of amputation. However, it should be noted that people who check their HbA1c levels twice or more per year are less vulnerable to amputation since they are more proactive regarding their health.

The odds of amputation for smokers was 3.44 times higher than for non-smokers. Yesil et al. [2] likewise showed that the OR of amputation associated with smoking was 1.42, which is consistent with our results (p=0.041).

In a prospective study by Yesil et al. [2] conducted in Turkey, patients who underwent amputation were much more likely to be affected by hypertension than patients who did not undergo amputation (p=0.018). Similarly, in our study, patients who underwent amputation were more likely to have high blood pressure. Our crude conditional logistic regression model found a relationship between amputation and hypertension that was not statistically significant. This finding may be attributed to the diet and lifestyle of the cases and controls, which was very comparable in both groups because they lived in the same regions. Moreover, the members of both groups had access to healthcare centers, making it possible that their blood pressure might have been actively managed.

In our study, conditional logistic regression analysis showed a significant relationship between higher BMI values and the risk of amputation. Each 1 kg/m2 increase in BMI was associated with a 1.20 times higher chance of amputation among the participants in this study, which was inconsistent with the results reported by Yesil et al. [2]. In their study, higher BMI values in patients with amputations did not increase the risk of amputation; on the contrary, BMI values were significantly lower in people with amputations (p=0.002). These findings might be attributable to the effects of the operations that these people had undergone.

Our study had some limitations. In some cases, patients who underwent amputation were reluctant to provide the relevant information. In some cases, they refused to participate in the study, and were replaced with alternatives.

Since the cases, who had been referred to Razi Hospital, were from a range of cities within Khuzestan province, it was difficult to collect the required data for the control group. In some cases, we collaborated with staff working in health centers or diabetes units in other cities to resolve this problem.

Another limitation of this study was the old age of the cases and controls. Problems such as low levels of literacy, poor vision, and hearing loss created difficulties in obtaining information through our questionnaire. In order to address this problem, we consulted the patients’ caregivers and asked them for help.

In our study, the most important factors that led to amputation in patients with diabetic foot ulcers were females, irregular monitoring of HbA1c levels, improper shoes, smoking, and a family history of diabetes. It is necessary to provide diabetic patients with proper information and to raise their awareness about the importance of using proper shoes, and it seems that healthcare centers can play an important role in preventing amputation in these patients. Hence, we recommend that the health authorities make corresponding plans. Finally, it seems that diabetic patients need to receive training in self-care. A comprehensive program designed by the authorities to provide such training might prevent many cases of amputation in diabetes patients.

## Figures and Tables

**Table 1.** Characteristics of the participants in the case and control groups

Variable	Case	Control	Univariate OR (95% CI)	p-value
Sex				
Male	91 (69.47)	121 (46.18)	1.00	
Female	40 (30.53)	141 (53.82)	2.62 (1.66, 4.11)	0.001
Age (yr)	62.26±8.16	62.58±8.14	1.08 (1.04, 1.12)	0.001
HbAlc screenings (yr)				
Twice or more	21 (16.03)	172 (65.65)	1.00	
Less than twice	110 (83.97)	90 (34.35)	8.5 (4.8, 15.04)	0.001
Smoking				
No	35 (26.72)	164 (62.60)	1.00	
Yes	96 (73.28)	98 (37.40)	4.26 (2.64, 6.87)	0.002
Family history of diabetes				
No	37 (28.24)	140 (53.44)	1.00	
Yes	94 (71.76)	122 (46.56)	2.88 (1.81, 4.58)	0.04
Insulin therapy				
Yes	79 (60.31)	100 (38.17)	1.00	
No	52 (39.69)	162 (61.83)	0.36 (0.23, 0.58)	0.03
Suitable shoes				
Yes	25 (19.08)	151 (57.63)	1.00	
No	106 (80.92)	111 (42.37)	4.75 (2.91, 7.76)	0.001
Occupation				
Farmer	39 (29.77)	66 (25.19)	1.00	
Employee	11 (8.40)	64 (24.43)	0.31 (0.15, 0.67)	0.003
Worker	35 (26.72)	29 (11.07)	1.95 (1.05, 3.63)	0.03
Self-employed	15 (11.45)	55 (20.99)	0.51 (0.26, 0.99)	0.05
Unemployed	31 (23.66)	48 (18.32)	0.99 (0.55, 1.82)	0.99
Education				
Illiterate	54 (41.22)	49 (18.70)	1.00	
Primary school	42 (32.06)	131 (50)	0.30 (0.17, 0.51)	0.001
High school diploma	27 (20.61)	60 (22.90)	0.41 (0.22, 0.75)	0.004
Academic degree	8 (11.6)	22 (8.40)	0.32 (0.13, 0.81)	0.02
Hypertension				
No	48 (36.64)	111 (42.37)	1.00	
Yes	83 (63.36)	151 (57.63)	1.26 (0.82, 1.92)	0.27
Insulin therapy (yr)	3.19±3.44	1.78±2.73	1.27 (1.02, 1.57)	0.001
Living with diabetes (yr)	8.7±4.2	8.3±4.1	1.49 (1.18, 1.89)	0.001
Body mass index (kg/m^2^)	26.8±3.37	25.61± 2.78	1.15 (1.07, 1.24)	0.001

Values are presented as number (%) or mean±standard deviation.

OR, odds ratio; CI, confidence interval; SD, standard deviation; HbA1c, hemoglobin A1c.

**Table 2.** The final model of the adjusted ORs of risk factors for amputation in diabetic patients, calculated using conditional logistic regression

Variable	OR	95% CI	p-value
Age	1.09	1.03, 1.17	0.006
Sex	8.66	2.68, 27.91	0.001
Infrequent HbA1c screenings	13.97	4.97. 39.26	0.001
Lack of suitable shoes	5.50	2.20, 13.77	0.001
Smoking	3.44	1.45, 8.13	0.005
Body mass index	1.20	1.03, 1.41	0.02
Employment			
Farmer	1.00		
Employee	0.18	0.04, 0.77	0.02
Worker	7.28	1.89, 28.02	0.004
Self-employed	0.52	0.16, 1.71	0.28
Unemployed	0.65	0.20, 2.13	0.47
Family history	3.96	1.49, 10.53	0.006

OR, odds ratio; CI, confidence interval; HbA1c, hemoglobin A1c.
